# Selective Hydrodemethylation of Methylalkylbenzenes on Potassium Hydride

**DOI:** 10.1002/anie.202521955

**Published:** 2025-12-24

**Authors:** Puyang Tian, Marc Figueras‐Valls, Fei Chang, Francesc Viñes, Francesc Illas, Alexey Fedorov

**Affiliations:** ^1^ Department of Mechanical and Process Engineering ETH Zürich Zürich CH‐8092 Switzerland; ^2^ Departament de Ciència de Materials i Química Física & Institut de Química Teòrica i Computacional (IQTCUB) Universitat de Barcelona c/ Martí i Franquès 1–11 Barcelona 08028 Spain

**Keywords:** KH catalysis, FT modeling, Hydrogen vacancy mechanism, Selective hydrodemethylation

## Abstract

Dealkylation and hydrodealkylation of alkylaromatics are key processes in the petrochemical industry. The typical susceptibility of alkyl groups to removal via both dealkylation and hydrodealkylation reactions in aromatic compounds follows the order CH_3_ < 1° < 2° < 3°, with methyl and tertiary alkyl groups having the slowest and fastest reaction rates, respectively. Here, we report the selective catalytic hydrodealkylation of methylalkylbenzenes using carbon‐supported potassium hydride (KH/C) under a H_2_ atmosphere, which exhibits an opposite reactivity pattern compared with that described above. This trend is observed across various methylalkylbenzenes and also aligns with the higher reactivity of toluene compared to other monoalkylbenzenes. Density functional theory calculations on *ortho*‐ethyltoluene as a model substrate rationalize the experimental results by indicating a lower rate‐limiting energy barrier for the direct removal of the methyl group as methane relative to the removal of the ethyl group as ethane on the (001) facet of KH. Our study reveals that the potassium hydride surface exhibits a distinct hydrodealkylation mechanism, characterized by selectivity for hydrodemethylation, which contrasts with that of zeolites, Mo‐ or Pt‐based hydrodealkylation catalysts, and homogeneous free‐radical pathways.

## Graphical abstract

Selective catalytic hydrodealkylation of methylalkylbenzenes on carbon‐supported potassium hydride is reported. The reaction exhibits a reactivity trend of 1° ∼ 2° ∼ 3° < CH_3_, which is opposite to traditional dealkylation and hydrodealkylation. DFT calculations on the (001) facet of KH with *ortho*‐ethyltoluene suggest that selectivity is controlled by the *ipso*‐H* attack from the KH surface, leading to the direct removal of CH_3_ as CH_4_ with a lower rate‐limiting barrier than the removal of C_2_H_5_.

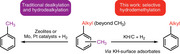

## Introduction

**Scheme 1:**
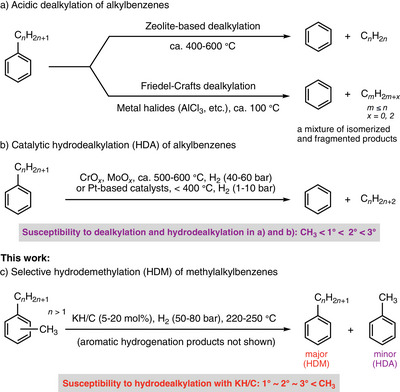
Approaches to converting alkylbenzenes into simpler aromatics (represented by benzene), including a) the zeolite‐based and Friedel–Crafts dealkylation, b) the transition‐metal‐catalyzed hydrodealkylation, and c) the selective hydrodemethylation of disubstituted methylalkylbenzenes developed in this work.

The synthesis of essential C_6_‐C_8_ aromatic petrochemicals such as benzene, toluene, and xylenes (i.e., the BTX fraction) from heavier mono‐ and dialkylbenzenes (C_9+_ hydrocarbons) is utilized industrially to align production with demand.^[^ ^1^, ^2^, ^3^ ^]^ A typical conversion methodology utilizes solid acid cracking catalysts such as zeolites that enable dealkylation, transalkylation, and disproportionation reactions of C_9+_ alkylbenzenes (Scheme 1a).^[^ ^1^, ^2^, ^3^ ^]^ Because carbenium‐ion‐like transition states are involved in these transformations, the susceptibility of the alkyl groups to react under solid acid catalysis follows the trend CH_3_ < 1° < 2° < 3°, which correlates with the stability of the respective alkyl carbenium species.^[^ ^4^, ^5^ ^]^ Indeed, the reaction rate for the conversion of alkylbenzenes on zeolites or amorphous aluminosilicates has been shown to increase from primary alkylbenzenes to secondary and tertiary alkyls.^[^ ^6^, ^7^ ^]^ This susceptibility of alkyl groups to dealkylation on solid acid catalysts is similar to that of Friedel–Crafts catalysts such as AlCl_3_ (Scheme 1a).^[^ ^8^, ^9^, ^10^, ^11^ ^]^

Despite a well‐established dealkylation process on solid acid catalysts, the petrochemical industry relies on hydrodealkylation (HDA) due to the higher selectivity, scalability, and robustness of this approach for upgrading heavy alkylbenzenes to the BTX fraction.^[^ ^12^, ^13^ ^]^ The HDA is a formal hydrogenolysis reaction of the C(*sp* ^2^)─C(*sp* ^3^) bonds of alkylaromatics with dihydrogen, typically under transition metal‐containing catalysts such as supported CrO*_x_* or MoO*_x_* at 500–600 °C and 40–60 bar H_2_, as shown in Scheme 1b.^[^ ^13^, ^14^, ^15^ ^]^ The susceptibility of alkylbenzenes to HDA has been compared over a Ni‐Mo/Al_2_O_3_ catalyst, revealing the trend parallel to that for dealkylation with solid acid catalysts: CH_3_ < Et < *i*‐Pr < *t*‐Bu.^[^ ^16^ ^]^ The observed trend has been attributed to the interaction energy of the alkylbenzene substrate with the electron‐deficient active site or the energetics of proton transfer to the aromatic intermediate.^[^ ^16^ ^]^ This difference in reactivity enabled the selective removal of higher alkyl groups from mixtures of methylbenzenes and alkylbenzenes. For instance, ethyl‐xylene and diethylbenzene are hydrodealkylated faster than trimethylbenzene, leading to a selective removal of the ethyl group on a catalyst containing H‐ZSM‐5 and noble metals (Pt, Pd, Rh) in mild conditions (380 °C, 1 bar).^[^ ^17^ ^]^

Under thermal (non‐catalytic) conditions, toluene hydrodemethylation (HDM) is performed under high temperatures of 700–900 °C, proceeding through a free‐radical mechanism.^[^ ^18^ ^]^ Since the bond dissociation energy (BDE) of the C(*sp* ^2^)─C(*sp* ^3^) bond is higher than that of the C(*sp* ^3^)─C(*sp* ^3^) side‐chain bonds in alkylbenzenes (ca. 103 and 77 kcal mol^−1^, respectively),^[^ ^19^ ^]^ alkylbenzenes are first cracked to toluene, followed by the HDM of toluene to benzene.^[^ ^13^ ^]^ Overall, selective hydrodemethylation of methylalkylbenzenes would not be in line with the free‐radical, carbenium ion, or electrophilic transition‐metal‐catalyzed hydrogenolysis mechanisms discussed above. To the best of our knowledge, selective HDM has not been developed.

Some of us have recently reported a HDM reaction that forms benzene from toluene at 250 °C under 50–80 bar of H_2_ with catalytic amounts of carbon‐supported potassium hydride (KH/C).^[^ ^20^ ^]^ Here, we studied the competitive removal of methyl and higher alkyl groups in monoalkylbenzenes and various methylalkylbenzenes under KH/C and H_2_ conditions. The results demonstrate that, in stark contrast to the susceptibility to solid acid dealkylation and both catalyzed and thermal hydrodealkylation, methylalkylbenzenes undergo selective HDM under the KH/C and H_2_ conditions (Scheme 1c). Density functional theory (DFT) calculations performed on *ortho*‐ethyltoluene, used as a model substrate, attribute this selectivity to the higher energy barrier leading to the removal of the ethyl group (as ethane) compared to the removal of the methyl group (as methane) from the key cyclohexadienyl intermediate on the (001) facet of KH.

## Experimental and Theoretical Aspects

### Experimental Details

KH/C with a nominal 30% weight loading of potassium was prepared by mixing a carbon support (graphite, surface area ca. 500 m^2^ g^−1^) with metallic potassium and exposing the resulting K/C material to 10 bar of H_2_ at 110 °C for 10 h. This method yields KH well dispersed on graphite, with a crystallite size estimated by the Scherrer equation of ca. 40 nm.^[^ ^20^, ^21^ ^]^ A representative procedure for the tests with KH/C was as follows. In an N_2_‐filled glovebox, an autoclave equipped with a glass liner was loaded with 50 mg of KH/C, 5 mL of dodecane, and the desired amount of substrate. The autoclave was sealed, removed from the glovebox, and connected to a high‐pressure H_2_ line. The line was purged three times with 100 bar H_2_, and the autoclave was pressurized to a set pressure of H_2_ and heated to the desired temperature using the ramping rate of 5 °C min^−1^. The stirring rate was 500 rpm. After the desired time, the autoclave was cooled to room temperature and depressurized. The reaction mixture was then exposed to air (except for the recycling experiments), filtered using filter paper, and the supernatant was collected. For the recycling experiments, 40 mg of KH/C and 4 mL of dodecane were used. The autoclave was purged with Ar and transferred into a N_2_‐filled glovebox. The supernatant was collected, and a new batch of starting material in dodecane was added for the subsequent test. A known amount of heptane internal standard was then added to the supernatant. The quantification was performed using gas chromatography‐mass spectrometry and a flame ionization detector (GC‐MS/FID).

Conversion (*X*) of a substrate (*A*) is calculated using the initial and final amounts of *A* (*N_A0_* and *N_A1_*, respectively), determined in the reaction mixture by GC analysis (Equation 1). The yield and selectivity of product P*_i_* (*Y_Pi_* and *S_Pi_*) are calculated using Equations 2 and 3, that is, the selectivity is based on the amounts of detected and quantified products. Selectivity among the products **2–8** is reported. The mass balance (M.b.) is calculated according to Equation 4.

(1) $$\;X_{A}=\frac{N_{A_{0}}\;-\;N_{A_{1}}}{N_{A_{0}}}\;\times 100\%$$

(2) $$\;Y_{P_{i}}=\frac{N_{P_{i}}}{N_{A_{0}}}\;\times 100\%$$

(3) $$\;S_{P_{i}}=\frac{N_{P_{i}}}{\sum_{j}N_{P_{j}}}\;\times 100\%$$

(4) $$M.\;b.=\frac{\sum_{j}N_{P_{j}}+N_{A_{1}}}{N_{A_{0}}}\;\times 100\%$$

### Computational Details

Bulk potassium hydride, the relevant surface, and its reactivity toward hydrodealkylation (i.e., both hydrodeethylation and hydrodemethylation) of *ortho*‐ethyltoluene have been studied using calculations based on the periodic density functional theory employing the Perdew–Burke–Ernzerhof (PBE) exchange‐correlation functional within the generalized gradient approach (GGA),^[^ ^22^ ^]^ with the effect of dispersion included using Grimme's D3 approach.^[^ ^23^ ^]^ The calculations were performed using the Vienna ab initio simulation package (VASP),^[^ ^24^ ^]^ in which the Kohn–Sham equations are solved by expanding the valence electron density in a plane‐wave basis set with a cutoff of 415 eV. The effect of core electrons on the valence region was included through the projected augmented wave (PAW) method as implemented by Kresse and Joubert.^[^ ^25^, ^26^ ^]^

KH exhibits a rock‐salt crystal structure corresponding to the cubic $\mathrm{Fm}\bar{3}m$ space group, where each potassium cation (formally K⁺) is surrounded by six equivalent hydride (formally H^−^) anions to form a mixture of corner and edge‐sharing KH_6_ octahedra.^[^ ^27^ ^]^ To analyze the reactivity towards HDA, we considered *ortho*‐ethyltoluene as a model substrate and the most stable surface, which for rock‐salt‐type solids is the (001) one.^[^ ^28^ ^]^ The lattice parameter and atomic structure of bulk KH have been determined using a minimum unit cell containing eight atoms. From the optimized bulk structure, stoichiometric KH (001) surface slab models were constructed, consisting of four atomic layers and a *c*(3×3) supercell, which is sufficiently large to accommodate the reacting species without significant interaction between the periodically repeated replicas. The atomic structure of the two bottommost layers is constrained to match the bulk structure, providing an appropriate environment for the two topmost layers with the fully optimized atomic structure. For the bulk and surface models, the necessary numerical integration in reciprocal space was carried out using a 15×15×15 Monkhorst‐Pack grid of special **k**‐points for bulk, and **Γ** point only for the surface model.^[^ ^29^ ^]^ The optimized lattice constant for bulk KH is determined to be 5.641 Å, which is in close agreement with the experimental cubic lattice parameter of 5.698 Å.^[^ ^30^ ^]^

To infer the most stable hydrogen coverage on the KH (001) surface under the experimentally relevant conditions (250 °C, 80 bar H_2_), we estimated the change in Gibbs free energy associated with the addition or removal of one hydrogen atom from a pristine KH surface, initially featuring full occupancy of hollow sites by hydrogen atoms (1 monolayer, ML H coverage). For the addition of hydrogen, all inequivalent adsorption sites were examined, with the most stable configuration identified as adsorption atop an already‐occupied hollow site. The Gibbs free energy differences between the various hydrogen coverages were calculated following Equations 5 and 6 below, where *G_pristine_* denotes the Gibbs free energy of the KH (001) surface model with full H coverage, i.e., where all hollow sites are occupied by H atoms, while *G*_*surface* + 1*H*_ denotes the Gibbs free energy of the same surface with an additional hydrogen atom adsorbed atop one of the already‐occupied hollow sites, *G*_*surface* − 1*H*_ denotes the Gibbs free energy of the surface with one hydrogen atom removed from a hollow site, and $G_{H_{2}}^{\left(g\right)}$ denotes the gas‐phase Gibbs free energy of the H_2_ molecule.

(5) $$\;\Delta G_{\mathrm{addi}\mathrm{tion}}=G_{\mathrm{surf}\mathrm{ace}+1H}\;-G_{\mathrm{pris}\mathrm{tine}}-\frac{1}{2}G_{H_{2}}^{\left(g\right)}$$

(6) $$\;\Delta G_{\mathrm{subt}\mathrm{ract}\mathrm{ion}}=G_{\mathrm{surf}\mathrm{ace}-1H}\;-G_{\mathrm{pris}\mathrm{tine}}+\frac{1}{2}G_{H_{2}}^{\left(g\right)}$$

The *G* values were estimated following the atomistic thermodynamics approach by Reuter and Scheffler.^[^ ^31^, ^32^ ^]^ Both Δ*G_addition_* and Δ*G_subtraction_* are positive, with values of 0.85 and 1.44 eV, respectively, indicating that the most stable hydrogen overlayer on the KH (001) surface under reaction conditions corresponds to 1 ML of hydrogen atoms occupying the hollow sites. For the surface reaction, the energy profiles for the reported mechanisms rely on the self‐consistently computed density functional total energy, rather than the Gibbs free energy, as entropic contributions are not expected to introduce significant energy changes to the potential energy surface.

The homogeneous free‐radical pathway was also studied using the VASPsol package.^[^ ^33^ ^]^ To provide an implicit solvent model for dodecane, which was used in experiments, a solvent permittivity of 2.01 was used.^[^ ^34^ ^]^ The reactants, transition state, and product atomic structures have been obtained from DFT calculations using the same approach as for the calculations with the KH (001) surface. Specifically, the atomic structures for the species of interest have been obtained either through suitable energy minimization for the minimum energy structures or, in the case of transition states (TSs), through the climbing‐image nudged‐elastic‐band (CI‐NEB) method, employing seven intermediate images. The acquired structures have been characterized, either as minima or transition states on the potential energy surface, through vibrational frequency analysis using Hessian matrix diagonalization. The Hessian matrix elements were obtained by finite differences of analytical gradients with atomic displacements of 0.015 Å. All reported minimum energy structures exhibit only positive eigenvalues, whereas the TS structures exhibit a single imaginary frequency.

The rate constants for the elementary steps in the homogeneous free‐radical pathway were subsequently approximated using conventional transition state theory (TST), according to:

(7) $$\;k_{TST}=\kappa \frac{k_{B}T}{h}e^{\left(-\frac{\Delta G^{‡}}{k_{B}T}\right)}$$

where *k_B_* is the Boltzmann constant, *h* is Planck's constant, *T* is the temperature, Δ*G* ^‡^ is the Gibbs free energy of activation, and κ is the transmission coefficient, assumed here to be one. This expression was used to evaluate the product selectivity in the two conceivable mechanisms, as discussed below. Specifically, the relative rates for the rate‐limiting steps of the free‐radical and adsorbate mechanisms were compared, taking into account entropy effects using Equation 7.

## Results

### Experimental Results

**Figure 1:**
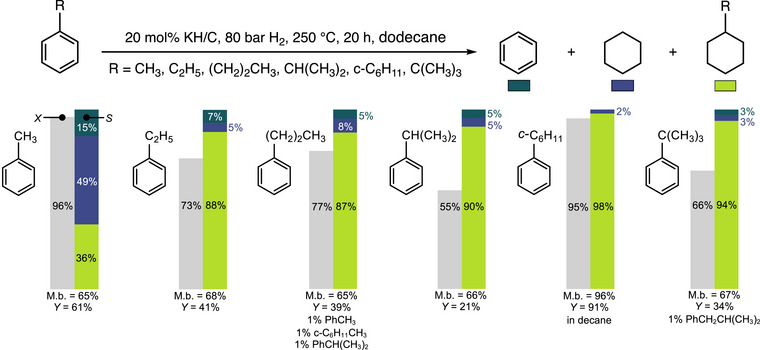
The hydrodealkylation of monoalkylbenzenes with KH/C and H_2_. M.b. denotes mass balance, *X* represents conversion, *Y* represents the combined yield of C_6_H_6_, *c*‐C_6_H_12_, and R‐*c*‐C_6_H_11_, and *S* is selectivity among these three products.

*Monoalkylbenzenes*: To assess the susceptibility of higher alkyl groups to removal using the KH/C and H_2_ methodology, we compared the yields of benzene and cyclohexane obtained from toluene, ethyl‐, *n*‐propyl‐, *i*‐propyl‐, cyclohexyl‐, and *t*‐butylbenzene after exposing their solutions in dodecane (0.375 M) to a 20 mol% KH/C (50 mg) and 80 bar H_2_ at 250 °C for 20 h. The GC quantification results are summarized in Figure 1, which presents the conversion of substrates and selectivity among the quantified hydrodealkylated and hydrogenated products, with complete details provided in Table S1. Under the mentioned conditions, toluene showed a 96% conversion, leading to benzene and cyclohexane in a combined 39% yield (calculated by multiplying the selectivity to benzene and cyclohexane by the total yield of all quantified products), with cyclohexane formed via the hydrogenation of benzene.^[^ ^20^ ^]^ The direct hydrogenation of toluene also provided methylcyclohexane in a 22% yield. Subjecting ethylbenzene and *n*‐propylbenzene to the same conditions gave only a 5% yield of benzene and cyclohexane, and a comparable yield of ethylcyclohexane and *n*‐propylcyclohexane (36% and 34%), although at lower conversions of 73% and 77%, respectively. The combined yield of benzene and cyclohexane decreased further to approximately 2% for *i*‐propylbenzene, cyclohexylbenzene, and *t*‐butylbenzene, obtained along with 19%, 89%, and 30% yields of the respective alkyl‐substituted cyclohexanes (conversions were 55%, 95%, and 66%, Table S1). Interestingly, *n*‐propylbenzene and *t*‐butylbenzene gave small amounts (about 1%) of isomerized substrates, i.e., *i*‐propylbenzene and *i*‐butylbenzene, as well as fragmented alkyl chain products (toluene and methylcyclohexane in the case of *n*‐propylbenzene).

These results demonstrate that, relative to toluene, both the conversion of monoalkylated benzenes and the yield of HDA products decrease appreciably for higher primary (C_2_H_5_, *n*‐C_3_H_7_), secondary (CH(CH_3_)_2_, *c*‐C_6_H_11_), and tertiary (C(CH_3_)_3_) alkyls. The high conversion in the case of cyclohexylbenzene is due to the hydrogenation to bicyclohexyl, which also correlates with a high mass balance (96%). This indicates that the adsorption of either starting alkylbenzenes or the reaction products onto KH/C in non‐polar dodecane likely contributes to the lower mass balance of 65%–68% for the other monoalkylbenzenes beyond cyclohexylbenzene. Additionally, a partial escape of low‐boiling benzene and cyclohexane into the headspace of the reactor can also reduce the mass balance (Figure S1). To assess this, 5 mL of a cumene solution in dodecane (0.375 M) was charged into the autoclave for a recovery test under reaction conditions (no KH/C was added), and 77% of the cumene was recovered. Overall, the susceptibility to hydrodealkylation obtained with monoalkylbenzenes under KH/C and H_2_ conditions is the opposite of the reported trends in acidic dealkylation and hydrodealkylation reactivity discussed above.

**Figure 2:**
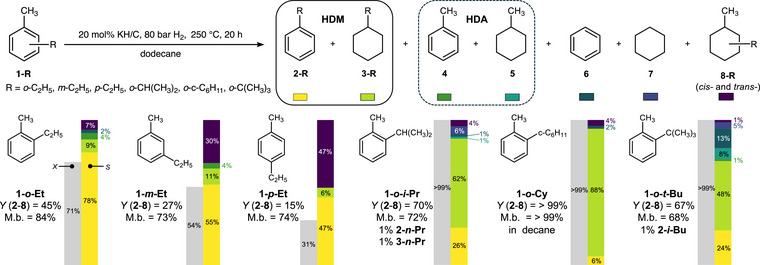
The selective hydrodemethylation of methylalkylbenzenes using KH/C and H_2_. The symbols *X*, *S*, and M.b. denote conversion, selectivity among products **2**–**8**, and mass balance, respectively.

*Methylalkylbenzenes*: Considering the results with monoalkylbenzenes, we envisioned that a selective HDM of disubstituted methylalkylbenzenes could proceed under the KH/C and H_2_ conditions. To evaluate this, we subjected *ortho‐*, *meta*‐, and *para*‐ethyltoluene (**1‐*o*‐Et**, **1‐*m*‐Et**, and **1‐*p*‐Et**, respectively) to the same hydrodealkylation conditions as utilized above. Substrates **1‐*o*‐Et**, **1‐*m*‐Et**, and **1‐*p*‐Et** gave the hydrodemethylation products ethylbenzene and ethylcyclohexane (**2‐Et** and **3‐Et**) in a combined 39%, 18%, and 8% yield and at the respective conversions of 71%, 54%, and 31% (Figure 2, Table S2). Interestingly, the hydrodealkylation products, toluene and methylcyclohexane (**4** and **5**), were obtained for both **1‐*o*‐Et** and **1‐*m*‐Et** at a notably lower combined yield not exceeding 2%–3%. Neither **4** and **5**, nor benzene and cyclohexane (**6** and **7**, formed due to the successive HDM/HDA reactions) were obtained with **1‐*p*‐Et**. However, **1‐*o*‐Et** and **1‐*m*‐Et** provided small amounts of **6** and **7** (1%–2% yield). The respective hydrogenation products of the starting isomeric ethyltoluenes (**8‐Et**, Figure 2) also formed in small amounts (3%, 8%, and 7% for **1‐*o*‐Et**, **1‐*m*‐Et**, and **1‐*p*‐Et**, respectively). Thus, the selectivity results with isomeric ethyltoluenes verify that hydrodemethylation proceeds faster than hydrodeethylation when using KH/C and H_2_. The higher conversion for the *ortho*‐substituted ethyltoluene relative to the *meta*‐ and *para*‐isomers is possibly linked to its higher intramolecular steric repulsion or weaker adsorption energy to the KH surface (vide infra).

To further explore the susceptibility of *ortho*‐substituted methylalkylbenzenes with secondary and tertiary alkyls to hydrodealkylation under our conditions, three additional substrates were studied, specifically, **1‐*o*‐*i*‐Pr**, **1‐*o*‐Cy**, and **1‐*o*‐*t*‐Bu** (Figure 2, Table S2). Under standard conditions, conversions greater than 99% were achieved for all three substates. Small amounts of direct hydrogenation products, denoted **8‐R** in Figure 2, were obtained for these substrates (4%, 4% and 1% for **1‐*o*‐*i*‐Pr**, **1‐*o*‐Cy**, and **1‐*o‐t*‐Bu**, respectively). **1‐*o*‐*i*‐Pr** gave HDM products isopropylbenzene and isopropylcyclohexane in a combined 61% yield. Only a 1% yield of HDA product **5** was obtained. Consecutive HDA/HDM of **1‐*o*‐*i*‐Pr** led to **6** and **7** in a combined yield of 5%; in addition, isomerized HDM products *n*‐propylbenzene and *n*‐propylcyclohexane were formed, both in a 1% yield. HDM of **1‐*o*‐Cy** produced cyclohexylbenzene and bicyclohexyl in a combined 94% yield. Only a trace amount of HDA product **5** was detected. Consecutive HDA/HDM of **1‐*o*‐Cy** gave 2% of **6** and a trace amount of **7**. A mass balance exceeding 99% was observed in the reaction of **1‐*o*‐Cy**, attributed to the low volatility and high steric hindrance of the reaction products, which lowered adsorption on KH/C. Subjecting **1‐*o‐t*‐Bu** to the same reaction conditions provided HDM products *tert*‐butylbenzene and *tert*‐butylcyclohexane in a combined yield of 48%. In addition, **1‐*o‐t*‐Bu** gave HDA products **4** and **5** in a combined 6% yield, along with a 1% yield of iso‐butylbenzene **2‐*i*‐Bu**. Consecutive HDM/HDA products (**6** and **7**) were obtained in the reaction of **1‐*o*‐*t*‐Bu** in a considerable 12% combined yield. Isobutane was detected in the reaction mixture of **1‐*o‐t*‐Bu** by GC‐MS/FID, consistent with the HDA reaction (Figure S7). Overall, all three additional substrates displayed higher conversion and HDM product yields compared with **1‐*o*‐Et**. The reason for this higher conversion may be attributed to the steric effect and the favorable difference in adsorption energy between starting materials and products on the KH surface (vide infra).

Lastly, using **1‐*o*‐*i*‐Pr** as a representative substrate, we verified that the preference for the hydrodemethylation over hydrodealkylation is independent of the specific reaction conditions. Thus, lowering the loading of KH/C from 20 to 10 mol% and to 5 mol% while keeping other parameters unchanged reduced the conversion of **1‐*o*‐*i*‐Pr** from > 99% to 91% (with 5 mol%) and slightly increased the selectivity of HDM from 85% to 90% (see Table S3 for details). The HDM selectivity is comparable (92%) when using 10 mol% KH/C at a lower temperature and pressure (50 bar, 220 °C, 20 h); these conditions yielded an 86% conversion of **1‐*o*‐*i*‐Pr**.

**Figure 3:**
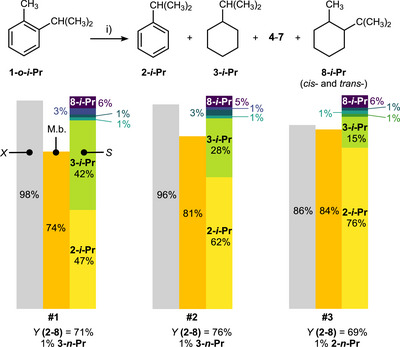
Recycling study using **1‐*o*‐*i*‐Pr** and KH/C (see Table S4 for details). Conditions i) were 20 mol% KH/C, 80 bar H_2_, 250 °C, 20 h. The symbols *X*, *S*, and M.b. denote conversion, selectivity among products **2**–**8**, and mass balance, respectively. See Figure 2 for the color code of all products.

The recyclability of KH/C was evaluated using **1‐*o*‐*i*‐Pr**. Its conversion gradually declined from over 99% with fresh KH/C (20 mol%) to 98%, 96%, and 86% over three recycling experiments, indicating appreciable recyclability (Figure 3, Table S4). Notably, the yield of the aromatic HDM product **2‐*i*‐Pr** increased during these experiments from 18% with fresh KH/C to 33%, 47%, and 52% in the first, second, and third cycles, respectively. This increase was offset by a decrease in the yield of the hydrogenated HDM product **3‐*i*‐Pr**, which declined from 44% with fresh KH/C to 30%, 21%, and 10% over subsequent cycles. The combined yield of HDM products (**2‐*i*‐Pr** and **3‐*i*‐Pr**) ranged from 62% to 68% while the combined yield of HDA products **4**–**5** was 1%–2%. Products **6**–**7** and **8** were also minor, accounting for 1%–3% and 3%–4%, respectively. The mass balance gradually increased with each KH/C recycling, from 74% to 81% and 84%, attributed to saturation of adsorption sites on KH/C by organic species. These adsorbed organics likely block sites involved in hydrogenation but do not significantly hinder the HDM‐active sites, leading to only a modest decline in conversion with KH/C recycling. Finally, a minor fraction of the reaction products can be lost to the autoclave headspace, as shown by a recovery experiment with **2‐*i*‐Pr** (vide supra), which contributed to the lower mass balance observed in this work.

Taken together, our experimental results demonstrate a more facile hydrodemethylation compared to the hydrodealkylation of higher alkyl groups in both monoalkyl and methylalkylbenzenes under the KH/C and H_2_ conditions. In the following, we present a theoretical study aimed at rationalizing the observed reactivity trends.

### Theoretical Modeling

*Free‐radical chain mechanism*: The experimental results revealed that small amounts of isomerized products, such as *i*‐butylbenzene, were observed when using *t*‐butyl‐substituted mono‐ and dialkylbenzenes. The formation of *i*‐butylbenzene is typical for a radical neophyl rearrangement, involving a 1,2‐phenyl shift (Scheme S1); other minor identified products are also consistent with radical isomerization pathways.^[^ ^35^, ^36^, ^37^, ^38^ ^]^ Such transformations may include hydrogen atom transfer (HAT) and hydrogen radicals (H⋅) formed in situ.^[^ ^39^, ^40^ ^]^ Considering these arguments, we first assessed the energy barriers in the free‐radical hydrodeethylation (HDE) and hydrodemethylation pathways using DFT calculations, with *ortho*‐ethyltoluene, **1‐*o*‐Et**, as a model substrate. Since dodecane was typically used experimentally as a solvent, it was simulated by an implicit model method, and thus without the explicit participation of the KH substrate.

**Figure 4:**
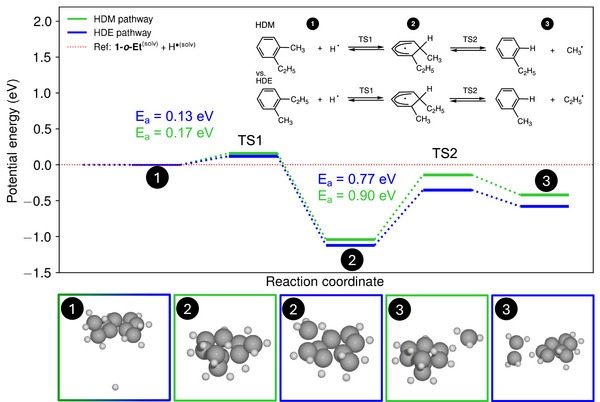
A computed free‐radical mechanism in dodecane comparing the hydrodemethylation and hydrodeethylation pathways for **1‐*o*‐Et** (presented in green and blue, respectively), with the structure of identified intermediates shown at the bottom. The intermediates are numbered as shown in the reaction scheme. Dark and light grey spheres represent carbon and hydrogen atoms, respectively.

The potential energy (i.e., the DFT total energy) profile shown in Figure 4 includes an attack of atomic hydrogen on one of the two *ipso*‐carbon atoms of **1‐*o*‐Et**, i.e., either bearing a methyl or ethyl group. This attack forms a cyclohexadienyl radical (a σ‐complex intermediate) through very low energy barriers, viz., 0.13 and 0.17 eV for the attack on the *ipso*‐carbon bearing ethyl and methyl group, respectively. These low barriers do not account for the energy required to form atomic hydrogen, which may form by replenishing a hydrogen vacancy (*V_H_*) at the KH (001) surface with H_2_. This process can be expressed as $H_{2}^{\left(g\right)}+V_{H}↔H_{L}+H^{*}$, where $H_{2}^{\left(g\right)}$ is a hydrogen molecule in the gas phase, *V_H_* denotes a surface hydrogen vacancy, *H_L_* denotes a healed surface (lattice) hydrogen vacancy, and H* denotes the hydrogen atom adsorbed atop the surface. The adsorbed H* atom can subsequently desorb as a free radical (i.e., atomic hydrogen). This mechanism requires 0.46 eV to dissociate H_2_ on a *V_H_* site, replenishing the vacancy and placing the second hydrogen as a surface adsorbate. Additionally, forming atomic hydrogen in solution by dissociation H* of the surface requires an overall of about 1.6 eV. The mechanism leading to a *V_H_* site is discussed below.

The selectivity‐determining kinetic barrier of the radical mechanism is the cleavage of the respective cyclohexadienyl radical to either the HDM or HDE product (TS2 in Figure 4). The energy barrier from the cyclohexadienyl radical to ethylbenzene (the HDM product) and methyl radical is 0.90 eV, which is higher than the 0.77 eV required for forming toluene (the HDA product) and ethyl radical. This result is in line with a higher C(*sp* ^2^)─C(*sp* ^3^) BDE in toluene relative to ethylbenzene, which are 103.9 and 102.3 kcal mol^−1^, respectively.^[^ ^41^ ^]^ To summarize, while the radical‐based processes can explain the presence of minor amounts of isomerized products, the experimentally observed preference for HDM over HDE, and, more generally, over HDA selectivity, is inconsistent with the free‐radical mechanism. We have therefore explored alternative pathways involving the KH surface to explain the experimental results.

*Surface adsorbate mechanism*. To identify the likely HDM pathway consistent with the experimental results, we considered an adsorptive mechanism on the KH surface. Toward this end, we first identified the most stable surface structure of KH under reaction conditions. Although several surface terminations of KH exist, it is reasonable to assume that, as with other rocksalt‐type solids, the most stable and predominant surface is the (001) facet,^[^ ^28^ ^]^ chosen in the present study. Next, we considered the hydrogen coverage on the KH surface under operating conditions (250 °C, a hydrogen partial pressure of 80 bar). At such a high hydrogen partial pressure, the KH (001) surface is expected not to contain hydrogen vacancies, which was indeed verified as discussed above. Thus, the most stable hydrogen overlayer on the KH (001) surface under reaction conditions corresponds to 1 ML of hydrogen atoms occupying the hollow sites. This result is consistent with the ionic nature of KH, where both K^+^ and H^ −^ ions possess filled valence shells, rendering the formation of additional chemical bonds energetically unfavorable, except for weak dispersion interactions.

**Figure 5:**
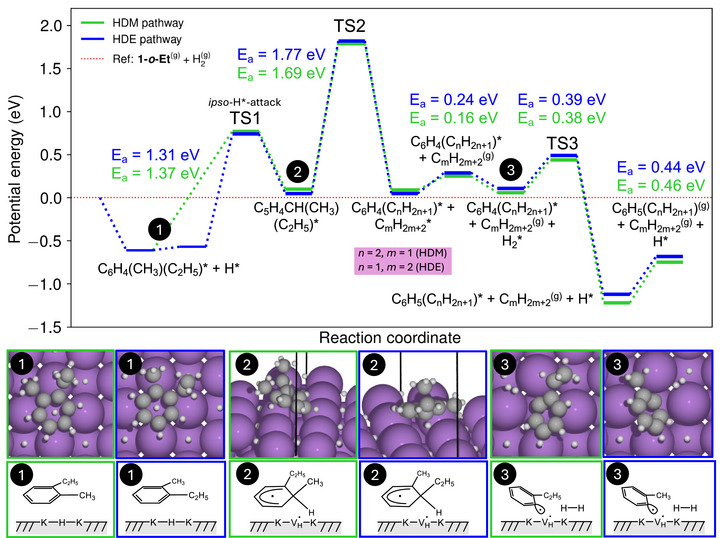
Reaction mechanism for the hydrodeethylation and hydrodemethylation of **1‐*o*‐Et** adsorbed onto the KH surface. The key intermediates, numbered and color‐coded as in the reaction pathway, are provided at the bottom. The vertical lines in the snapshots of the modeled intermediates 2 are the unit cell vectors. Note that in state 3, an H_2_ molecule from the environment is adsorbed onto the surface. Purple, grey, and white spheres represent potassium, carbon, and hydrogen atoms, respectively. The sizes of the spheres are chosen for clarity and are not to scale with the respective atomic or ionic radii.

The elementary steps for the HDE and HDM of **1‐*o*‐Et** were investigated as illustrated in Figure 5. The reaction begins with the adsorption of the **1‐*o*‐Et** molecule on the KH (001) surface. The most stable adsorption configuration places the molecular center of mass above a hollow site, with the methyl group oriented toward an adjacent bridge site. This configuration is depicted in Figure 5 (bottom, state 1). Although the HDM and HDE mechanisms are conceptually analogous, the ethyl group removal pathway includes an additional step that involves rotating the **1‐*o*‐Et** molecule to position the ethyl group above a bridge site. This intermediate is only 0.04 eV less stable than the most favorable adsorption geometry, and thus easily reachable. Following this reorientation, both mechanisms align the respective alkyl group (methyl or ethyl) above a bridge site, enabling interaction with a hydrogen atom located in the hollow site directly beneath the molecule. This facilitates hydrogen transfer to the *ipso* carbon (bearing either a methyl or an ethyl group), forming the C_5_H_4_CH(CH_3_)(C_2_H_5_) intermediate (Figure 5, bottom, state 2). Hydrogen transfer to other aromatic carbon atoms was also examined, and although transfer to the *ortho* position was found to yield a slightly more stable intermediate than to the *ipso* position (Table S5), no plausible mechanism was established to connect such intermediates with the hydrodealkylation pathway. Consequently, the only feasible outcome of the *ortho* transfer is reversion to the initial state.

For both alkyl groups, the subsequent step involves the highest activation energy and thus constitutes the rate‐limiting step. This step consists of the simultaneous cleavage of the C(sp^3^)─C(sp^3^) and C(sp^3^)─H bonds in the cyclohexadienyl intermediate, where the H atom connected to the *ipso* carbon forms a new bond with the methyl or ethyl group to yield methane or ethane, respectively, along with yielding the respective aryl radical species. Note that this is a distinct C─H bond‐forming step that has not been found in the free‐radical mechanism discussed above. Interestingly, the surface vacancy created by the subtraction of hydrogen interacts with the radical‐bearing carbon of the aryl ring, stabilizing this intermediate. Notably, our attempts to locate the transition states for the cleavage of methyl or ethyl radicals of the KH‐adsorbed cyclohexadienyl intermediate, i.e., similar to the TS2 in the free‐radical pathway (Figure 4), were unsuccessful and converged to alkane elimination (methane or ethane) already discussed above. This result underlines the importance of the Ar⋅⋅⋅*V_H_* interaction in stabilizing the aryl radical species and, consequently, TS2 in Figure 5.

Our attribution of the aryl species formed after TS2 and found interacting with the KH (001) surface as radical (in preference to anionic) has the following supporting evidence. First, starting from the pristine KH (001) surface with 1 ML hydrogen coverage, where all hollow sites are occupied, the computed energies associated with the ejection of hydrogen into the gas phase as a radical (H·) and as a hydride anion (i.e., leaving in this case a positively charged surface point defect) are 3.08 eV and 3.45 eV, respectively, indicating that ejection as a radical is thermodynamically preferred. Our second argument is based on the electronic character of the hydrogen atom involved in the *ipso* attack in TS1 (Figure 5). A Bader charge analysis carried out for TS1 (HDM pathway) indicated the net charge of the reacting hydrogen atom of 1.495 *e*, which is lower than the 1.70 *e* net charge found for a reference hydrogen atom in a hollow site. Since hydrogen atoms in the KH surface sites are hydridic, the reduction in net charge for the transferring hydrogen atom in TS1 supports a radical‐type *ipso*‐attack.

Lastly, to close the catalytic cycle, the weakly bound methane or ethane products desorb from the surface. In addition, to restore the KH surface and form the aromatic product, a gas‐phase H_2_ molecule adsorbs near the radical aryl intermediate and dissociates in a concerted step, simultaneously occupying the radical site on the aromatic ring and replenishing the hydrogen vacancy on the surface. This step forms either toluene or ethylbenzene (depending on the reacting alkyl group).

## Discussion

In the following, we briefly consolidate experimental and computational results with the main mechanistic insights. It is worth noting that, although both HDE and HDM adsorbate pathways of **1‐*o*‐Et** modeled by DFT exhibit similar activation barriers, the reaction selectivity is governed by a single rate‐limiting step that defines the overall reaction kinetics. Specifically, this step corresponds to TS2 in Figure 5, leading to the concerted elimination of either methane or ethane from the respective cyclohexadienyl intermediate. Based on the obtained DFT results, the rate‐limiting step exhibits activation energies of 1.69 eV and 1.77 eV for the methyl and ethyl groups, respectively. This small but noticeable difference in energy barriers has a considerable impact on the exponential term of the rate expression and allows explaining the experimental observations, where hydrodemethylation of **1‐*o*‐Et** is favored. Taking into account the temperature and entropy effect by explicit calculation of the Gibbs free energy for this step, and using transition state theory to approximate the rates of the limiting concerted elimination of either the methane or ethane group according to Equation 7, leads to the rate at 250 °C for the HDM being about 70 times faster than that of HDE. This result is in reasonable agreement with experimental findings. In contrast, the rate for the HDE according to the mechanism in Figure 4 would be expected to be about 11 times faster than that of HDM at 250 °C, which is not observed experimentally. That being said, we cannot exclude the possibility that the homogeneous free‐radical mechanism contributes to the formation of the HDE product. At the same time, we can exclude the selective formation of the HDM product by the free‐radical mechanism. Taken together, these results provide compelling evidence for the adsorbate mechanism.

**Scheme 2:**
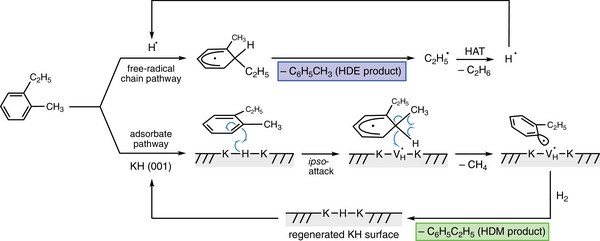
Proposed mechanisms selective for HDM (adsorbate pathway on the KH surface) or HDE (free‐radical pathway). Atomic hydrogen to initiate the free‐radical pathway may be generated by healing a hydrogen vacancy on the KH surface with H_2_.

The selectivity‐determining transition state that favors HDM over HDE in the decomposition of the surface cyclohexadienyl intermediate involves two key differences with respect to the homogeneous free‐radical pathway. These two differences are entangled. That is, the presence of the nearby *V_H_* site, formed after the *ipso*‐attack, allows for the stabilization of the aryl species after the elimination of methane (or ethane) from the cyclohexadienyl radical. This additional stabilization (not available in the free‐radical pathway) shifts the elimination pathway from forming the alkyl radical (observed in the free‐radical pathway) to creating the alkane (observed in the adsorbate pathway). Scheme 2 summarizes these results.

Additionally, we note that graphite, used in this work to support and disperse KH, has a π‐type HOMO.^[^ ^42^, ^43^ ^]^ Its interaction with the highly ionic KH is likely of the charge‐induced dipole type. While this interaction may impact the surface atoms of KH in direct contact with the support, the exterior KH sites accessible to reactants are expected to remain highly ionic. This suggests that the support has minimal influence on the HDM and HDA mechanisms. Indeed, our periodic KH model effectively captures the observed selectivity trends. Therefore, we conclude that the main role of graphite is to stabilize KH nanoparticles against sintering through a weak charge‐induced dipole interaction.

## Conclusion

We have reported a selective hydrodemethylation of methylalkylbenzenes that uses a KH/C catalyst under 50–80 bar H_2_ and 220–250 °C. The established susceptibility of hydrodealkylation under the KH/C and H_2_ conditions is 1° ∼ 2° ∼ 3° < CH_3_, which contrasts with that of traditional zeolite, Friedel–Crafts, and hydrodealkylation catalysts, as well as radical processes (CH_3_ < 1° < 2° < 3°). The higher reactivity of the methyl group relative to higher alkyls is explained by DFT modeling of *o*‐ethyltoluene, which reveals a lower transition state energy for methane than for ethane elimination on the KH (001) facet. Such a step is unavailable in the solution‐phase radical pathway because the resulting aryl radical species are stabilized by the surface hydrogen vacancy. These findings provide a rare example of a reaction catalyzed by the KH surface, exhibiting selectivity inaccessible through alternative routes.

## Conflict of Interests

The authors declare no conflict of interest.

## Supporting information



Supporting Information

## Data Availability

The computational data is available upon request; all other data are provided in the Supporting Information (SI) file, which contains experimental procedures, product characterization, and computational details.
