# Supplementary material for: Selective Hydrodemethylation of Methylalkylbenzenes on Potassium Hydride
Source: Angew Chem Int Ed Engl. 2025 Dec 24;65(6):e21955. doi: 10.1002/anie.202521955 (PMC12865151; doi:10.1002/anie.202521955)
Supplement: Supplementary file 1 — Supporting Information [file ANIE-65-e21955-s001.pdf]

Supporting Information

Selective Hydrodemethylation of Methylalkylbenzenes on Potassium Hydride

Puyang Tian,[1#] Marc Figueras-Valls,[2#] Fei Chang,[1] Francesc Viñes,[2*] Francesc Illas,[2] Alexey Fedorov[1*]

[#] These authors contributed equally.

[1] Department of Mechanical and Process Engineering, ETH Zürich, CH-8092 Zürich, Switzerland

[2] Departament de Ciència de Materials i Química Física & Institut de Química Teòrica i Computacional (IQTCUB), Universitat de Barcelona, c/ Martí i Franquès ,1-11 08028 Barcelona, Spain

E-mail: francesc.vines@ub.edu, fedoroal@ethz.ch

Table of Contents

Methods **2**

General experimental 2

Quantification of product yields by GC-FID analysis 3

Figures **4**

Figure S1. Autoclaves from Premex Solutions GmbH used in this work. 4

Figure S2. GC-MS chromatogram of the reaction mixture with **1-*p*-Et**. 5

Figure S3. GC-MS chromatogram of the reaction mixture with **1-*m*-Et**. 5

Figure S4. GC-MS chromatogram of the reaction mixture with **1-*o*-Et** 6

Figure S5. GC-MS chromatogram of the reaction mixture with **1-*o*-*i*-Pr** 6

Figure S6. GC-MS chromatogram of the reaction mixture with **1-*o*-Cy** 7

Figure S7. GC-MS chromatogram of the reaction mixture with **1-*o*-*t*-Bu** 7

Tables **8**

Table S1. Hydrodealkylation of monoalkylated benzenes with KH/C 8

Table S2. Selective hydrodemethylation of methylethylbenzenes. 9

Table S3. Selective hydrodemethylation of *ortho*-isopropyltoluene 9

Table S4. Recycling study using **1-*o*-*i*-Pr** and KH/C. 10

Table S5. Reaction energy differences for the surface H transfer to various aromatic carbons of the absorbed **1-*o*-Et** intermediate. 10

Schemes **11**

References **12**

Methods

General experimental

The following chemicals were purchased from the indicated vendors and used as received (solids) or were degassed by three freeze-pump-thaw cycles before use (liquids): metallic potassium (98%, Sigma Aldrich), graphene nanoplatelets (quality level 100, Sigma Aldrich), cyclohexane (99%, Sigma Aldrich), benzene (99%, Sigma Aldrich), methylcyclohexane (99%, Acros Organics), toluene (99.5%, Sigma Aldrich), ethylcyclohexane (99%, TCI), ethylbenzene (99%, TCI), propylcyclohexane (98%, TCI), propylbenzene (99%, TCI), isopropylcyclohexane (99%, TCI), cumene (99%, TCI), bicyclohexyl (99%, Sigma Aldrich), phenylcyclohexane (97%, Sigma Aldrich), *tert*-butylcyclohexane (99%, TCI), *tert*-butylbenzene (99%, Sigma Aldrich), 2-ethyltoluene (99%, Sigma Aldrich), 3-ethyltoluene (99%, Sigma Aldrich), 4-ethyltoluene (95%, Sigma Aldrich), *o*-cymene (98%, BLD Pharmatech), 1-cyclohexyl-2-methylbenzene (98%, BLD Pharmatech), 2-*tert*-butyltoluene (99%, TCI), heptane (99%, Sigma Aldrich), decane (99%, TCI) and dodecane (99%, TCI).

Synthesis of KH/C and the subsequent reactions were performed in a high-pressure autoclave from Premex Solutions GmbH (volume 60 ml; maximum temperature 250 °C; maximum pressure 250 bar), equipped with a mechanical stirrer and operated *via* an Ordino CS350 controller (Figure S1).

Identification and quantification of products was performed using a Perkin-Elmer Clarus 560 S gas chromatography mass spectrometer/flame ionization detector (GC-FID/MS) equipped with a (5%-phenyl)-methylpolysiloxane capillary column (Elite-5). Oven temperature program used in a typical GC-MS analysis included holding for 5 min at 40 °C and then heating to 170 °C with a ramp rate of 10 °C min^{-1}. Heptane was added to the reaction mixture as an internal standard after the reaction. The retention times of internal standard and major products obtained using this GC program are: benzene (3.6 min), cyclohexane (3.7 min), heptane (4.5 min), methylcyclohexane (5.2 min), toluene (6.4 min), ethylcyclohexane (8.3 min), ethylbenzene (8.9 min), isopropylbenzene (10.4 min), cumene (10.4 min), propylcyclohexane (10.5 min), propylbenzene (11.0 min), *tert*-butylcyclohexane (11.7 min*), tert*-butylbenzene (11.8 min), bicyclohexyl (16.5 min) and phenylcyclohexane (16.7 min). Typical gas chromatograms of the reaction mixture after selective HDM tests of methylalkylbenzenes are shown in Figures S2-S7. GC peaks of benzene and cyclohexane, cumene and isopropylcyclohexane, *tert*-butylcyclohexane and *tert*-butylbenzene partially overlap. Quantification of benzene, cyclohexane, cumene, isopropylcyclohexane, *tert*-butylcyclohexane, and *tert*-butylbenzene was performed by GC-MS following a method described by us previously.[1]

Quantification of product yields by GC-FID analysis

The yields of direct hydrogenation products of methyl alkylbenzenes were quantified using GC-FID. In this case, the molar response factors (MRF) were calculated employing the method of Chaintreau et. al.:[2, 3]

Relative to mesitylene, a hydrocarbon compound C_xH_y with *z* benzene rings has an $MRF_{C_xH_y}$ defined as:

$$MRF_{C_xH_y} = \frac{-61.5+88.8\times9+18.7\times12+127}{-61.5+88.8x+18.7y+127z} \qquad \text{(Eq. 1)}$$

The concentration of C_xH_y is calculated according to:

$$C_{C_xH_y} = MRF_{C_xH_y} \times C_{mesitylene} \times \frac{A_{C_xH_y}}{A_{mesitylene}} \qquad \text{(Eq. 2)}$$

where $A_{C_xH_y}$ and $A_{mesitylene}$ are the peak areas of compound C_xH_y and mesitylene, respectively. $C_{C_xH_y}$ and $C_{mesitylene}$ are the concentration of C_xH_y and mesitylene, respectively.

Figures

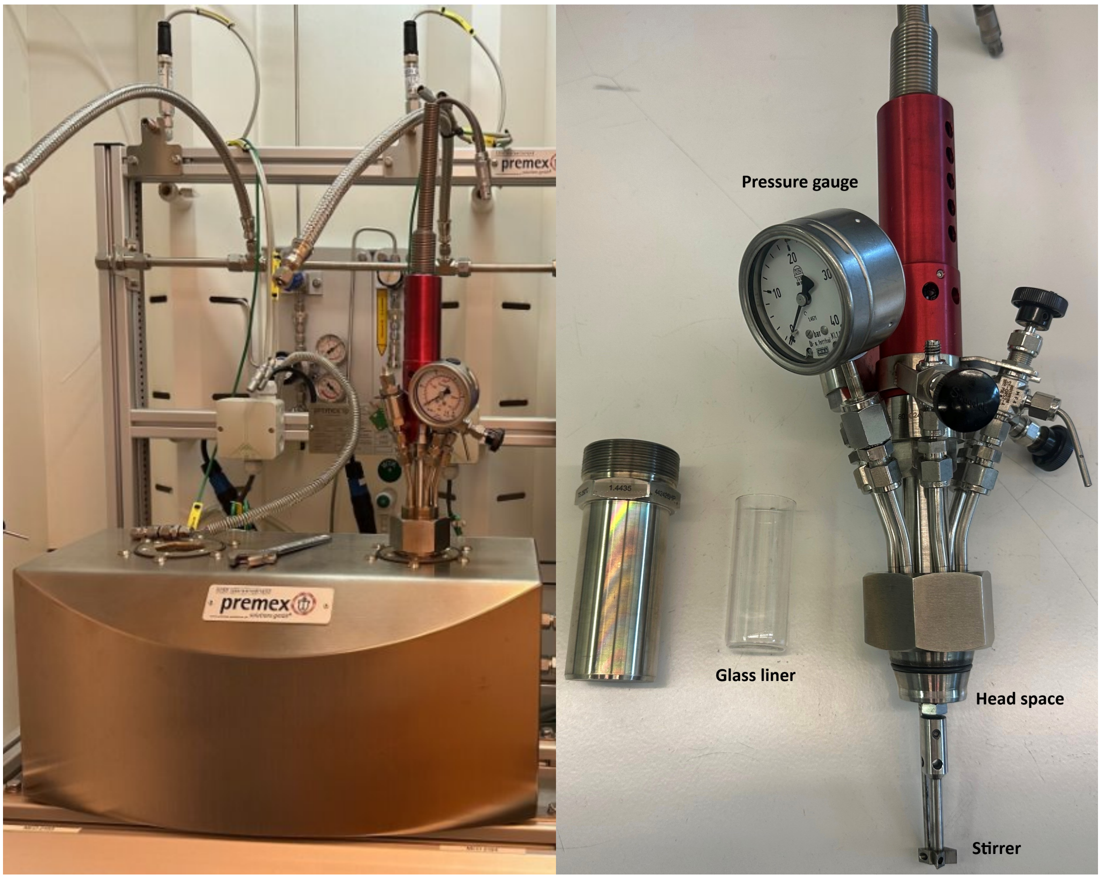

Figure S1. Autoclaves from Premex Solutions GmbH used in this work.

For the GC traces presented in Figures S2-S7, reaction conditions were: 250 °C, 80 bar H_2, 20 h, 1.875 mmol of a substrate, 50 mg of KH/C in 5 ml of dodecane.

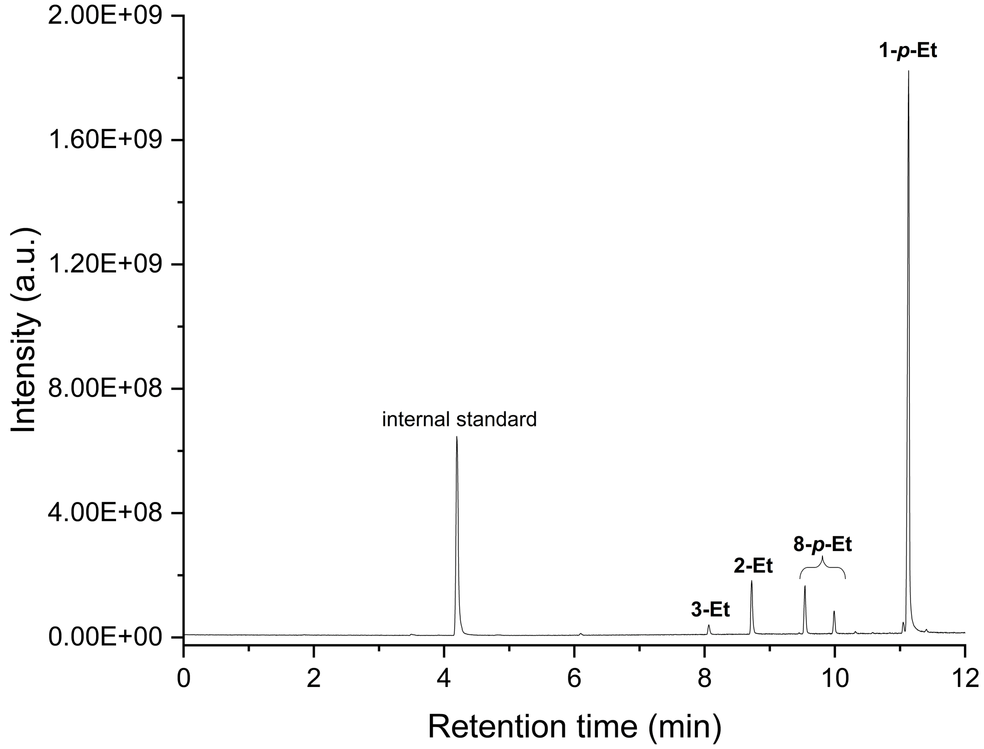

Figure S2. GC-MS chromatogram of the reaction mixture with **1-*p*-Et**.

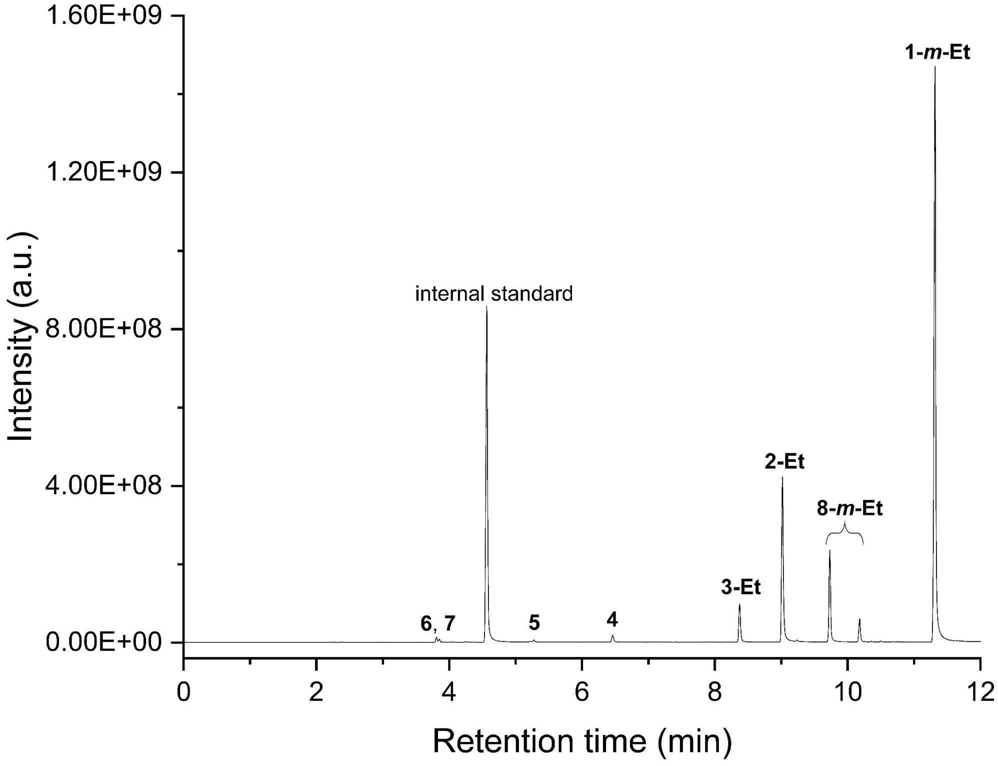

Figure S3. GC-MS chromatogram of the reaction mixture with **1-*m*-Et**.

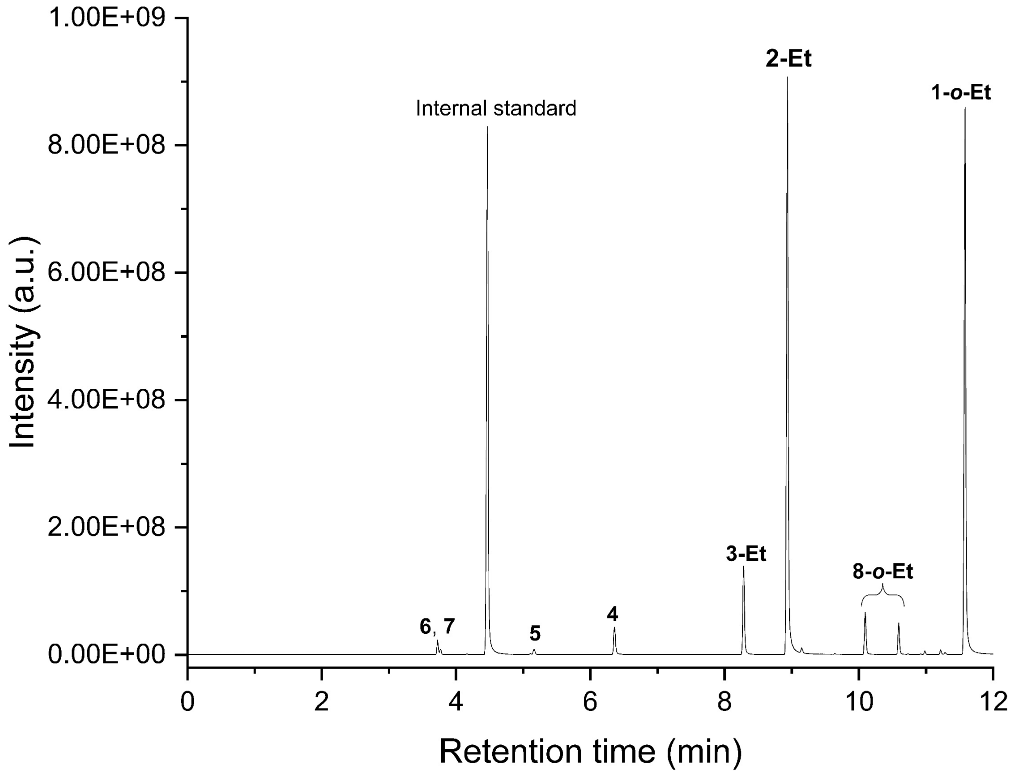

Figure S4. GC-MS chromatogram of the reaction mixture with **1-*o*-Et**.

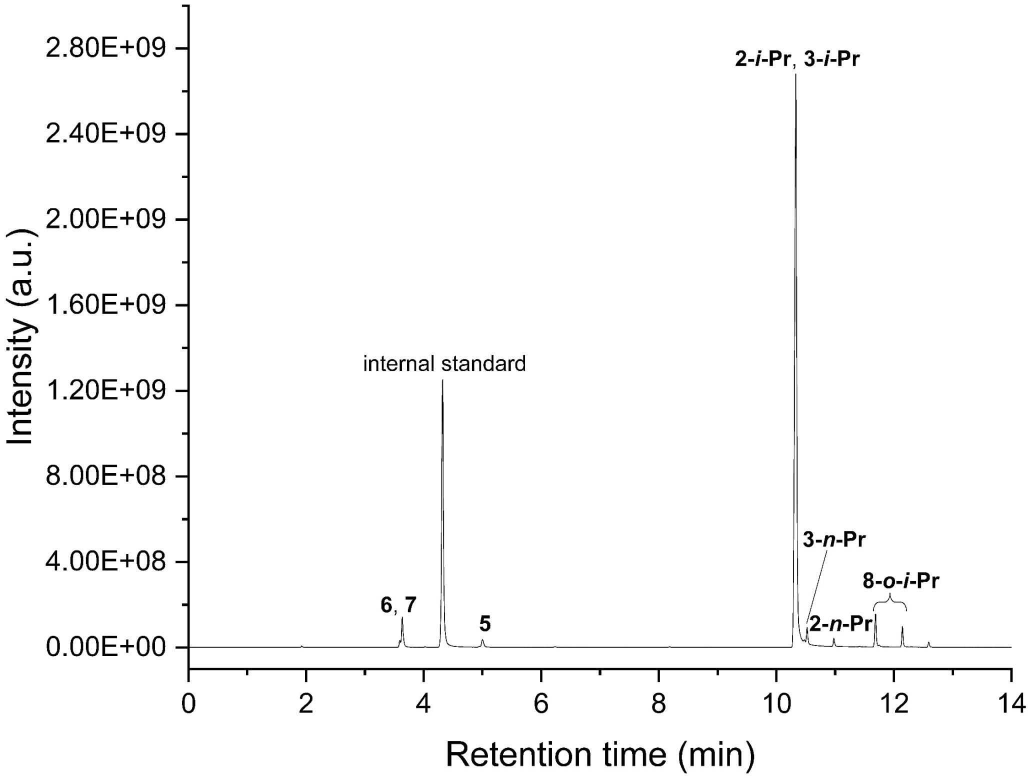

Figure S5. GC-MS chromatogram of the reaction mixture with **1-*o*-*i*-P**r.

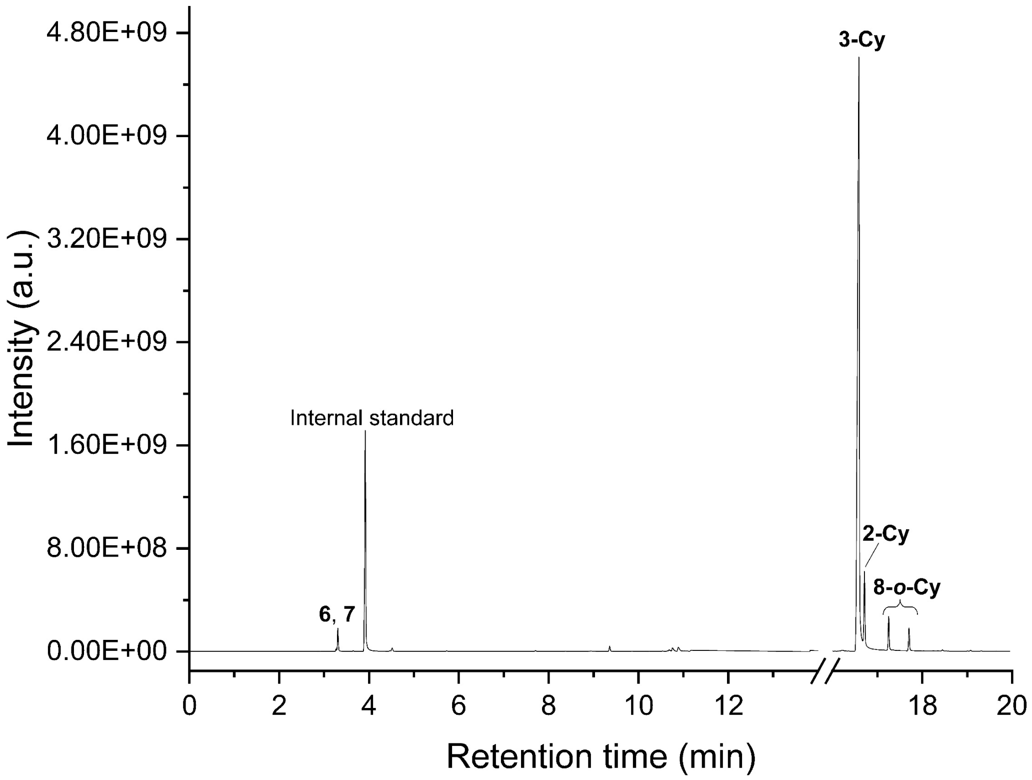

Figure S6. GC-MS chromatogram of the reaction mixture with **1-*o*-Cy**.

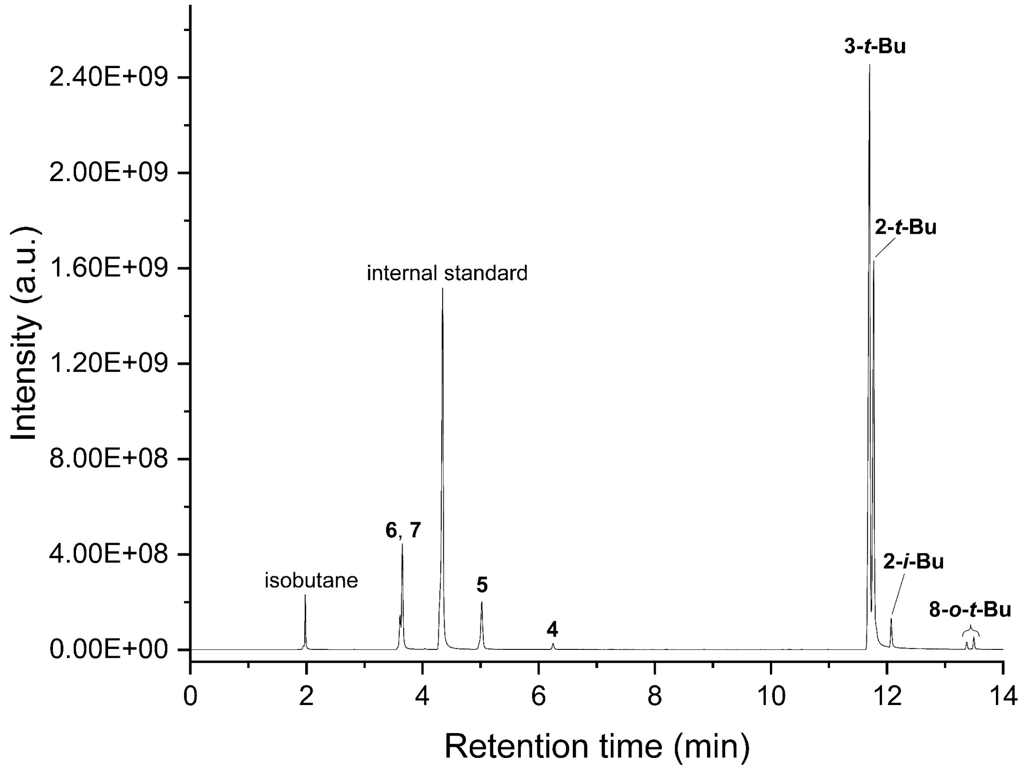

Figure S7. GC-MS chromatogram of the reaction mixture with **1-*o*-*t*-Bu**.

Tables

Table S1. Hydrodealkylation of monoalkylated benzenes with KH/C.

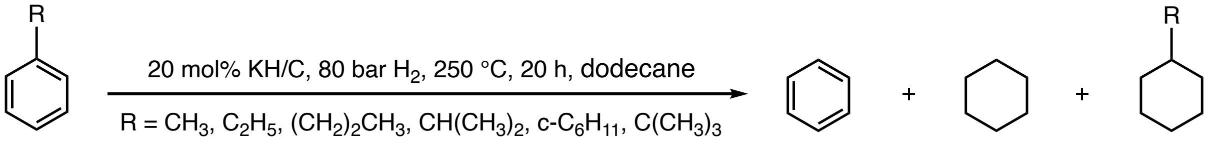

Entry	R in R–Ph	*X* (%)	Product yield / selectivity (%)[a]			Mass balance (%)
			C_6H_6 (%)	c-C_6H_{12} (%)	R–*c*-C_6H_{11} (%)	
1	CH_3	96	9/15	30/49	22/36	65
2	C_2H_5	73	3/7	2/5	36/88	68
3[b]	$(CH_2)_2CH_3$	77	2/5	3/8	34/87	65
4	$CH(CH_3)_2$	55	1/5	1/5	19/90	66
5[c]	c-C_6H_{11}	95	<1/0	2/2	89/98	96
6[d]	$C(CH_3)_3$	66	1/3	1/3	30/94	67

[a] Selectivity is calculated based on C_6H_6, *c*-C_6H_6 and R-*c*-C_6H_{11} [b] 1% of $PhCH_3$, 1% of *c*-$C_6H_{11}CH_3$ and 1% of *i*-C_3H_7–Ph formed. [c] Reaction was conducted in decane (5 mL). The yields of C_6H_6 and *c*-C_6H_{12} were divided by 2 to reflect the reaction stoichiometry. [d] 1% of *i*-C_4H_9-Ph formed.

Table S2. Selective hydrodemethylation of methylethylbenzenes.

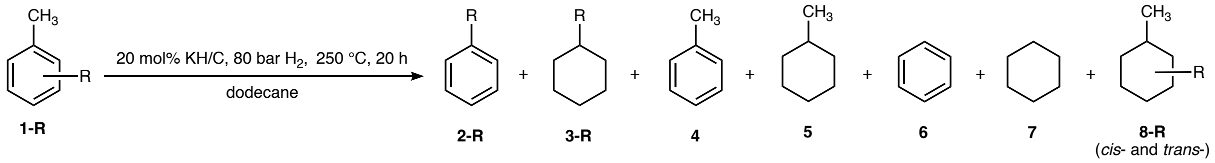

R = *o*-C_2H_5, *m*-C_2H_5, *p*-C_2H_5, *o*-$CH(CH_3)_2$, *o*-c-C_6H_{11}, *o*-$C(CH_3)_3$

Entry	**1-R**	*X* (%)	Product yield / selectivity (%)							Mass balance (%)
			**2-R**	**3-R**	**4**	**5**	**6**	**7**	**8-R**	
1	**1-*o*-Et**	71	35/78	4/9	2/4	<1/0	1/2	<1/0	3/7	74
2	**1-*m*-Et**	54	15/55	3/11	1/4	<1/0	<1/0	<1/0	8/30	73
3	**1-*p*-Et**	31	7/47	1/6	0/0	0/0	0/0	0/0	7/47	84
4[a]	**1-*o*-*i*-Pr**	>99	18/26	43/62	0/0	1/1	1/1	4/6	3/4	72
5[b]	**1-*o*-Cy**	> 99	6/6	88/88	0/0	<1/0	2/2	<1/0	4/4	> 99
6[c]	**1-*o*-*t*-Bu**	> 99	16/24	32/48	1/1	5/8	9/13	3/5	1/1	68

[a] 1% of **2-*n*-Pr** and 1% of **3-*n*-Pr** formed. [b] Reaction was conducted in decane (5 mL). The yields of **6** and **7** were divided by 2 to reflect the reaction stoichiometry. [c] 1% of **2-*i*-Bu** formed.

Table S3. Selective hydrodemethylation of *ortho*-isopropyltoluene.

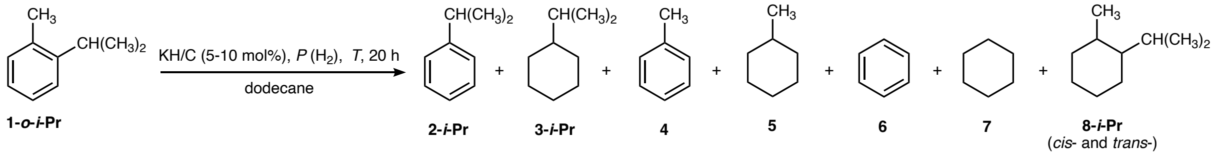

Entry	KH/C (mol %)	*X* (%)	*P* (bar)	*T* (°C)	Product yield / selectivity (%)							Mass balance (%)
					**2-*i*-Pr**	**3-*i*-Pr**	**4**	**5**	**6**	**7**	**8-*o*-Pr**	
1[a]	10	> 99	80	250	32/49	26/39	<1/0	1/1	1/1	3/5	3/5	68
2	5	91	80	250	45/78	7/12	2/3	<1/0	1/2	<1/0	3/5	67
3	10	86	50	220	44/84	4/8	1/2	<1/0	1/2	<1/0	2/4	66

[a] 1% **2-*o*-*n*-Pr** and 1% **3-*o*-*n*-Pr** found.

Table S4. Recycling study using **1-*o*-*i*-Pr** and KH/C.

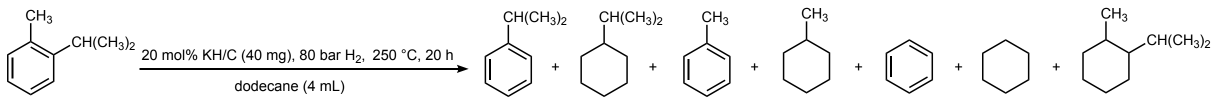

1-*o*-*i*-Pr **2-*i*-Pr** **3-*i*-Pr** **4** **5** **6** **7** **8-*i*-Pr** (*cis*- and *trans*-)

Entry	Recycled time	*X* (%)	Product yield / selectivity (%)							Mass balance (%)
			**2-*i*-Pr**	**3-*i*-Pr**	**4**	**5**	**6**	**7**	**8-*i*-Pr**	
1[a]	0	>99	18/26	44/62	< 1/0	1/1	1/1	4/6	3/4	72
2[a]	1	98	33/47	30/42	< 1/0	1/1	1/1	2/3	4/6	74
3[a]	2	96	47/62	21/28	< 1/0	1/1	2/3	1/1	4/5	81
4[b]	3	86	52/76	10/15	1/1	1/1	1/1	< 1/0	4/6	84

[a] < 1% **2-*o*-*n*-Pr** and 1% **3-*o*-*n*-Pr** found. [b] 1% **2-*o*-*n*-Pr** and < 1% **3-*o*-*n*-Pr** found.

Table S5. Reaction energy differences for the surface H transfer to various aromatic carbons of the absorbed **1-*o*-Et** intermediate.

Numbering in **1-*o*-Et** used	Aromatic carbon position	Reaction Energy (eV)[a]
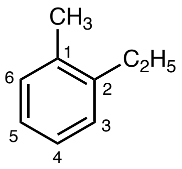	2	−0.05
	3	+0.05
	4	+0.09
	5	+0.07
	6	+0.04

[a] Referenced to the hydrogen transfer to the *ipso* position bearing the CH_3 group.

Schemes

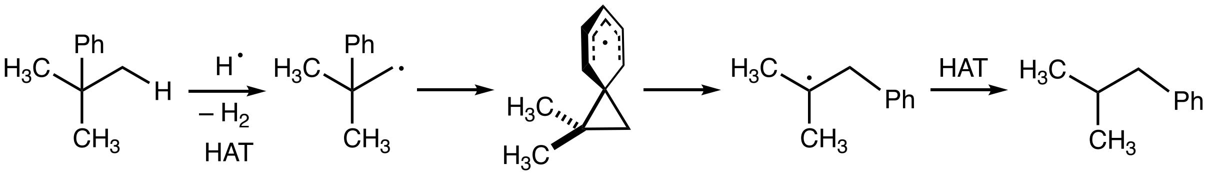

Scheme S1. A radical neophyl rearrangement explaining the formation of minor amounts of isomerized products such as isobutylbenzene formed from tert-butylbenzene.[4-6]

HAT stands for hydrogen atom transfer.

References

(1) Chang, F.; Fedorov, A. Production of Benzene by the Hydrodemethylation of Toluene with Carbon-Supported Potassium Hydride. *ChemSusChem* **2023**, *16*, e202202029.
(2) de Saint Laumer, J.-Y.; Cicchetti, E.; Merle, P.; Egger, J.; Chaintreau, A. Quantification in Gas Chromatography: Prediction of Flame Ionization Detector Response Factors from Combustion Enthalpies and Molecular Structures. *Anal. Chem.* **2010**, *82*, 6457-6462.
(3) Tissot, E.; Rochat, S.; Debonneville, C.; Chaintreau, A. Rapid GC-FID quantification technique without authentic samples using predicted response factors. *Flavour Fragr. J.* **2012**, *27*, 290-296.
(4) Franz, J. A.; Barrows, R. D.; Camaioni, D. M. Arrhenius Parameters for Rearrangements of the Neophyl, 1-Indanylmethyl, 2-Allylbenzyl, and 2-(2-Vinylphenyl)ethyl Radicals Relative to Hydrogen Abstraction from Tributylstannane. *J. Am. Chem. Soc.* **1984**, *106*, 3964-3967.
(5) Newcomb, M. Radical Kinetics and Clocks. In *Encyclopedia of Radicals in Chemistry, Biology and Materials*, Chatgilialoglu, C., Studer, A. Eds.; Vol. 1; John Wiley & Sons, Ltd, 2012; pp 107-124.
(6) Asensio, A.; Dannenberg, J. J. Phenyl-Bridging in the 2-Phenylethyl Radical. A Molecular Orbital Study. *J. Org. Chem.* **2001**, *66*, 5996-5999.
